# Haematopoietic Stem Cell Transplant Trends in Pakistan: Activity Survey from Pakistan Bone Marrow Transplant Group

**DOI:** 10.1155/2023/8865364

**Published:** 2023-09-28

**Authors:** Natasha Ali, Raheel Iftikhar, Muhammad Ayaz Mir, Syed Waqas Bokhari, Jehanzeb Ur Rehman, Uzma Zaidi, Shahzad Nasir, Salman Naseem Adil, Tariq Satti, Qamar Un Nisa Chaudhry, Muhammad Farhan, Tasneem Farzana, Tariq Ghafoor, Bushra Ahsan, Azhar S. Khan, Farrukh Ali Khan, Syeda Itrat Fatima, Shafaq Abdul Samad, Aliya Batool, Hafiz Muhammad Nadeem, Syed Nasir Abbas Bukhari, Saqib Hussain Ansari, Parvez Ahmed

**Affiliations:** ^1^Aga Khan University, Karachi, Pakistan; ^2^Armed Forces Bone Marrow Transplant Center, Rawalpindi, Pakistan; ^3^Shifa International Hospital, Islamabad, Pakistan; ^4^Shaukat Khanum Memorial Cancer Hospital, Lahore, Pakistan; ^5^National Institute of Blood Diseases, Karachi, Pakistan; ^6^Quaid-e-Azam International Hospital, Islamabad, Pakistan; ^7^Gambat Institute of Medical Sciences, Gambat, Pakistan; ^8^The Children's Hospital Lahore, Lahore, Pakistan; ^9^Dow University of Health Sciences, Karachi, Pakistan; ^10^Pakistan Institute of Medical Sciences, Islamabad, Pakistan; ^11^Akbar Niazi Teaching Hospital, Islamabad, Pakistan; ^12^Children's Hospital Karachi, Karachi, Pakistan

## Abstract

Pakistan is the fifth most populous country with a population of 225 million and has health expenditure accounting for only 2.8 percent of gross domestic product (GDP). Accordingly, there are a limited number of haematology-oncology and transplant centers in the country. The Pakistan Blood and Marrow Transplant (PBMT) group was established in 2020, and this report is the first activity survey from January 2021 to December 2022 focusing on the trends of matched-related donor, haploidentical, and autologous transplants in a developing country. A total of 12 transplant centers contributed data on the modified PBMT survey form retrospectively and 806 haematopoietic stem cell transplants (HSCTs) were carried out during the study duration. Allogeneic HSCT constituted 595 (73.8%) of all the transplants; this is in stark contrast to Western data, where autologous HSCT accounts for the majority of transplants. *ß*-thalassemia major and aplastic anemia were the commonest indications for allogeneic HSCT, in contrast to Western data, where acute leukemia is the leading transplant indication. Autologous transplants were more frequently performed for Hodgkin's lymphoma as compared to non-Hodgkin's lymphoma and multiple myeloma. The use of peripheral and bone marrow stem cells was comparable. A myeloablative conditioning regimen was routinely used in patients with acute leukemia. This report provides an insight of HSCT trends in Pakistan which are different from those of Western centers contributing to transplant data from South Asia.

## 1. Introduction

Haematopoietic stem cell transplant (HSCT) has been actively performed for the treatment of various haematological disorders since its discovery in 1957 [1]. In Pakistan, the HSCT program was initiated in 1995 [2], and in the span of over two decades, there are currently 12 operational centers which offer this facility. As of 2021, Pakistan's population is estimated to be around 225 million [3]. With a growth rate of 2.4% per year, Pakistan is the world's fifth most populous country. Accordingly, Pakistan's gross national income (GNI) per capita is estimated to be around $1,600. This is lower than the global average GNI per capita of $10,204, and it is also significantly lower than the per capita of other South Asian countries, such as India, which has a GNI per capita of $6,829 along with Bangladesh (GNI of $2,820) and Sri Lanka (GNI of $3,610). Pakistan spends 2.8% of its GDP on healthcare which is significantly less as compared to the developed world [4]. The cost of a stem cell transplant procedure in Pakistan depends upon the transplant center (public or private), type of transplant, conditioning regimen used, and posttransplant complications. It varies from $8000 to $25,000 across different centers. The cost of an allogeneic transplant is relatively higher due to donor matching and posttransplant care. The 12 operational centers are distributed in two provinces of the country i.e., Punjab and Sindh. These centers receive patient referrals from within and outside of Pakistan. However, based on our healthcare infrastructure, socioeconomic disparities affect patients' ability to afford medical treatments.

The Pakistan Blood and Marrow Transplant (PBMT) group was established in February 2020 to allow healthcare professionals and scientists in Pakistan involved in blood and marrow transplantation and related cellular therapies to collaborate and participate in promoting high-quality transplant services in the country. It has representation from all stem cell transplant centers and aims to promote and share current knowledge of all aspects of HSCT to establish, maintain, and promote the highest standards of care. The group commenced its first survey of HSCT activities in 2021 and was continued in 2022.

This yearly activity will become an instrument used to observe the trends in transplant indications and transplant type, to update conditioning protocols, graft versus host disease (GvHD) prophylaxis, and infection prevention according to local and international guidelines. The survey will also compare transplant activities in Pakistan from Western countries and discuss reasons for disparity.

## 2. Materials and Methods

### 2.1. Data Collection and Definitions

This was a retrospective observational survey based on the data of HSCTs performed in 2021 and 2022, collected from 12 transplant centers in the country. The survey tool collected data on the main indication and stage of the disease for which a transplant was performed (leukemias, lymphoproliferative disorders, solid tumours, bone marrow failure syndromes, haemoglobinopathy, primary immunodeficiency syndromes, and other inherited disorders). Based on the type of transplant (allogeneic or autologous), information on the type of conditioning regimen used was also incorporated. Other variables were stem cell source and donor type. The number of HSCTs was counted as 2 in patients who had received transplants twice. Similarly, if a patient received HSCT using more than one donor source, the case was counted as a mixture that contained bone marrow (BM) and peripheral blood (PB) stem cells. We also collected data on haploidentical donor defined as a first-degree relative (parent, child, and sibling) with 2 or more loci mismatched at human leucocyte antigen (HLA) A, B, C, DRB1, and DQB1 in the graft versus host and/or host versus graft direction.

### 2.2. Participating Teams and Data Managers

The 12 teams and data managers who have contributed to the survey are located mainly in two provinces of the country. These include Armed Forces Bone Marrow Transplant Center (AFBMTC), Shaukat Khanum Memorial Cancer Hospital (SKMCH), Shifa International Hospital (SIH), Quaid-e-Azam International Hospital (QIH), Pakistan Institute of Medical Sciences (PIMS), Akbar Niazi Teaching Hospital (ANTH), the Children's Hospital Lahore (CHL), from the province of Punjab and Aga Khan University (AKU), National Institute of Blood Diseases (NIBD), Gambat Institute of Medical Sciences (GIMS), Dow University of Health Sciences (DUHS), and Children's Hospital Karachi (CHK) from the province of Sindh.

### 2.3. Ethical Approval and Statistical Analysis

Since no individual patient data were used, ethics committee approval was not mandated. Each center signed a data sharing agreement prior to the collection of information. All descriptive variables were analyzed using Excel and Statistical Package for the Social Sciences (SPSS) version 22.

## 3. Results

**Figure 1:**
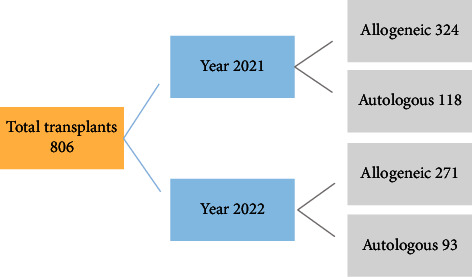
Summary of total transplants from 2021 to 2022.

**Figure 2:**
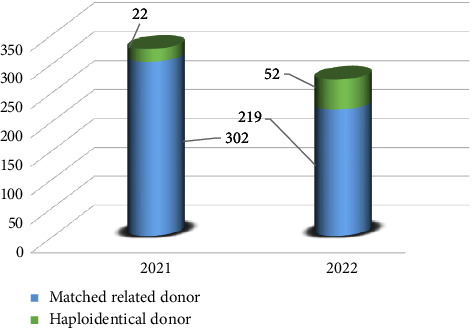
Summary of allogeneic transplants from 2021 to 2022.

The number of allogeneic and autologous transplants performed from 2021 to 2022 across Pakistan was 595 and 211, respectively. The total match-related donor transplants were 521, while haploidentical procedures were 74 (Figures 1 and 2).

From 2021 to 2022, the number of transplants performed in AFBMTC was 249, SKMCH was 96, SIH was 132, QIH was 56, PIMS was 23, ANTH was 14, CHL was 37, AKU was 55, NIBD was 73, GIMS was 30 DUHS was 24, and in CHK was 9. Five centers have been performing haploidentical transplants regularly while the rest were inclined towards match-related donors.

**Figure 3:**
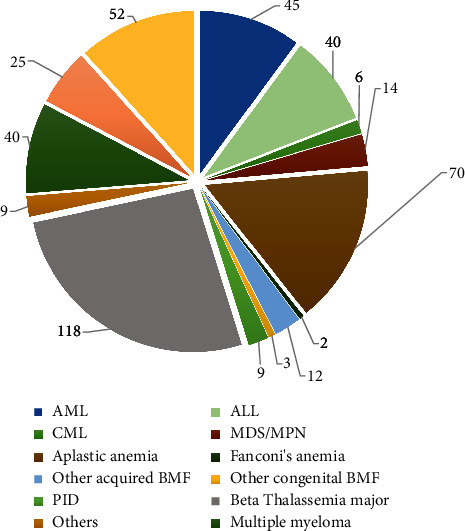
Indications of transplants in 2021.

In 2021, transplants for acute myeloid leukemia (AML) in first complete remission (CR) were performed in 34 patients and in relapsed AML, 12 patients. Similarly, for acute lymphoid leukemia (ALL), the transplant procedure for patients in first complete remission was carried out in 30 patients and for subsequent remission in 10 patients. In 2021, approximately 70 transplants were performed for aplastic anemia, while 118 procedures were carried out for *β*-thalassemia major (*β*TM). For multiple myeloma, 40 procedures were performed while, for non-Hodgkin's (NHL) and Hodgkin's lymphoma (HL), 25 and 52 patients underwent autologous transplants, respectively, in 2021. The rest of the transplant indications are given in Figure 3.

**Figure 4:**
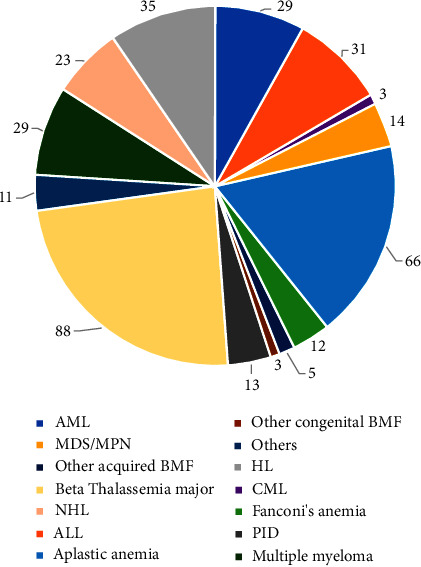
Indications of transplants in 2022.

We performed 19 transplants for AML in the first CR and 10 for the subsequent CR in 2022. For ALL, 21 and 10 transplants were performed for patients in the first and subsequent CR. Sixty-six procedures were carried out for aplastic anemia and 88 for *β*TM in 2022. Twelve procedures were also carried out for Fanconi's anemia, while 10 patients underwent the procedure for myelodysplastic syndrome (MDS). For NHL and HL, 23 and 55 autologous transplants were carried out, respectively, and in 29 patients, transplants were completed for the diagnosis of multiple myeloma. The rest of the transplant indications are given in Figure 4.

Bone marrow stem cells (BMSCs) as graft source were used in 380 patients, and in 407 patients, peripheral blood stem cells (PBSCs) were used. A bone marrow (BM) and peripheral blood (PB) stem cell mixture was used in 19 patients mainly in benign disorders (aplastic anemia, *β*TM) and haploidentical transplants.

A myeloablative regimen was used in 199 patients with various haematological disorders. Reduced-intensity conditioning was given to 33 patients while 38 patients received nonmyeloablative chemotherapy.

## 4. Discussion

This report describes number and types of transplants carried out across Pakistan from 2021 to 2022. When compared with the EBMT activity survey report of 2017, the main indications for transplants were myeloid malignancies, lymphoid malignancies, and solid tumours. The trend of previous years showed that the majority of HSCTs for lymphoid malignancies were autologous, while most transplants for myeloid malignancies were performed using stem cells from healthy donors [5]. In Pakistan, the main indications for HSCT are *β*-thalassemia major and aplastic anemia. Because of the large family structure, the possibility of finding related matched donors is potentially increased serving as a critical factor in opting for allogeneic transplantation. For a very long time, there were a total of three transplant centers operating in the country, but in the last decade, this number has increased to 12, and all centers have participated in this survey. ßTM is one of the most prevalent haemoglobinopathies in Pakistan [6], with a carrier rate of 5% [7]. Approximately 40,000 children are registered for transfusion services, and 5,000–9,000 children are born with this disorder annually [8]. With limited life expectancy and transfusion resources, haematopoietic stem cell transplant remains to be the only curative option in these patients. A previous experience published by Ullah et al. [9] has demonstrated an overall survival (OS) of approximately 80%. Nevertheless, *β*-thalassemia major remains to be the most common indication of allogeneic stem cell transplant in Pakistan, and the trend in the last two years has remained the same as 206 transplants were performed.

In developing countries, the management of patients with aplastic anemia remains challenging as there are impediments in diagnosis, unavailability of drugs, and delay in referrals of patients to tertiary care centers for management. In Pakistan, an estimated 3.5 patients/million population is diagnosed with aplastic anemia every year [10]. Approximately one-third of these patients can receive stem cell transplant as a cure. Fludarabine-based conditioning along with antithymocyte globulin (ATG) and cyclophosphamide has emerged as a promising alternate regimen with excellent results where Chaudhry et al. [11] and Zaidi et al. [12] have reported an OS of 84% and 76%, respectively. As evident in the literature, the prevalence of aplastic anemia in the Asian population is 3 times higher as compared to Western countries with environmental contaminants such as benzene, arsenic, and other pesticides providing an increased predisposition in the country [13]. Therefore, in Pakistan, aplastic anemia is the second most common benign haematological indication for allogeneic stem cell transplant as shown in Figures 3 and 4. Transplants performed for other acquired (e.g., paroxysmal nocturnal haemoglobinuria) and congenital bone marrow failure syndromes (e.g., Fanconi's anemia) were 23 in the last two years. There are limited studies on this group of diseases in Pakistan, but suffice to say that based on its prevalence in the highly intermingled and consanguineous cultural background of the country, it can be hypothesized that the true incidence may be much higher than quoted in literature [14].

The efficacy of allogeneic stem cell transplant for acute leukemias was confirmed in the 1990s through prospective trials that compared long-term outcomes in various subgroups undergoing the procedure with matched sibling donor [15, 16]. A meta-analysis of 24 trials with 6007 patients with acute myeloid leukemia showed a significant overall survival benefit for poor-risk (HR, 0.73; 95% CI, 0.59–0.90) and intermediate-risk AML (HR, 0.83; 95% CI, 0.74–0.93) in first complete remission [17]. Similarly, we completed HSCT in over 50 patients with AML in their first CR using a myeloablative regimen during the study period. We performed a total of 71 transplants for ALL (first and subsequent remissions combined) from 2021 to 2022. Genetic factors were not documented in the complete registry. However, the main indications were high-risk or relapsed ALL. The Swedish acute leukemia registry has reported that overall survival for patients <60 years of age is very similar for AML and ALL (42% 5-year OS and 38% 8-year OS), even though patients with ALL are younger and achieve CR1 faster than patients with AML. However, the OS in AML in first CR is greater than ALL especially in patients less than 55 years [18]. Our cohort mostly consisted of the same age group, i.e., <55 years for both ALL and AML.

Chemotherapy remains to be the first-line standard of care for aggressive lymphomas. However, approximately 30% of patients with NHL fail to achieve CR with standard chemotherapy regimens and require autologous stem cell transplants for cure [19, 20]. In contrast, Hodgkin lymphoma (HL) is a highly treatable malignancy with cure rates ranging from 85% to 90% of early-stage patients and 70% to 80% for advanced-stage patients. For the portion of patients who have relapse/refractory disease, randomized trials have established salvage chemotherapy followed by high-dose chemotherapy with autologous haematopoietic stem cell transplant (AHCT) as the standard of care leading to improved progression-free survival (PFS) [21]. In Pakistan, according to a report published in 2008, the frequency of NHL was 73% making it more common than HL with diffuse large B cell lymphoma (DLBCL) being the commonest subtype. During the study period, we performed 48 AHCT for NHL and 87 transplants for HL. The emerging molecular and prognostic characterization of DLBCL has changed the treatment paradigm in recent years with improvements in chemotherapy protocols, dose intensification of chemotherapeutic agents, and introduction of the second generation of immunotherapies leading to much better rates of CR and PFS [22]. This may be the reason for the decreased NHL frequency of patients undergoing AHCT in our cohort. Nevertheless, a total of 125 transplants were carried out for lymphomas making it the most common indication of an autologous transplant in our study.

Multiple myeloma (MM) accounts for 10% of all haematological malignancies [23]. Major developments in the treatment of MM over the past couple of decades have led to continued improvement in survival. Standard-risk patients receive at least four cycles of triplet/quadruplet regimens followed by AHCT and lenalidomide maintenance [24]. AHCT is an integral component of myeloma management and has contributed to survival improvement in the past two decades as several randomized trials and meta-analyses have confirmed that transplant is associated with deepening of the response rate and improvement in progression-free survival [25, 26]. Approximately 70 transplants were performed for MM during the study period making it the second most common indication for AHCT.

Currently, the trend in all transplant centers has shown an increased tendency towards PBSCs as graft sources owing to faster engraftment and ease of collection despite a higher rate GvHD and decreased survival rates in patients with nonmalignant disorders [27]. On the other hand, BMSCs are the preferred graft source in benign haematological disorders, especially aplastic anemia. In our part of the region, these recommendations may not completely apply due to late presentation of the patients suffering from this disease and the heavy transfusion load leading to increased graft failure rates [28]. Since 45% of our transplants were performed for benign haematological disorders, our use of both graft sources was almost similar. In a few patients of aplastic anemia and haploidentical procedures, we used a combination of PBSCs and BMSCs.

Myeloablative conditioning (MAC) regimens allow rapid engraftment of donor cells which may be followed in a proportion of patients by GvHD. The risk of transplant-related mortality (TRM) with MAC has decreased overtime due to improved HLA matching technology and supportive care [29]. Patients with leukemia have benefitted most from these improvements. In 70% of our patients with leukemia, we used a myeloablative conditioning regimen.

This report has several limitations, mainly reflecting that the data are from a retrospective, observational registry. PBMT has collected data of only those patients who underwent stem cell transplant; therefore, patients who were transplant eligible but could not undergo the procedure cannot be addressed. Secondly, we have not collected information on outcomes with respect to GvHD, transplant-related infections, progression-free survival, and overall survival. Still, this report provides directions towards further data collection processes which can be enhanced, and more parameters will be included for future analysis.

## 5. Conclusion

In summary, 806 transplant procedures were performed during the study period. *β*-thalassemia major and aplastic anemia were the common indications for allogeneic transplants while autologous transplant was more frequently performed for Hodgkin's lymphoma. PBSCs and BMSCs were comparable while myeloablative conditioning regimen was routinely used in transplants for acute leukemia. This report provides an insight of HSCT trends in Pakistan which are different from those of Western centers contributing to transplant data from South Asia.

## Data Availability

The data that support the findings of this study are available from the corresponding author upon reasonable request.

## Abbreviations

HSCT: Haematopoietic stem cell transplant
PBMT: Pakistan Blood and Marrow Transplant
ALL: Acute lymphoid leukemia
AML: Acute myeloid leukemia
CML: Chronic myeloid leukemia
MDS/MPN: Myelodysplastic syndrome/myeloproliferative neoplasm
PID: Primary immunodeficiency
NHL: Non-Hodgkin's lymphoma
HL: Hodgkin's lymphoma
BMF: Bone marrow failure.
